# Fainting

**Published:** 1891-07

**Authors:** 


### FAINTING.

Every person should know how to treat one in a faint. The following information may be found of value in some unexpected emergency.

When any one faints he should be placed in a recumbent position, with his head low, if he is pale and bloodless, but high, if red in the face, and every tight-fitting garment should be loosened. Then he should be fanned in the open air or by a window, cold water should be sprinkled over him, and his temples bathed with vinegar, ether, or cologne, while ammonia, burnt feathers, or singed hair are held beneath his nose, and his nostrils are tickled to make him sneeze. If the faint be a deep one, an enema of vinegar may be administered, the feet and hands bathed in warm water, the soles of the feet chafed, and mustard applied over the heart. Upon coming out of the faint the patient should still preserve for a time the reclining or recumbent position, and should drink a little cold water, some brandy and water in the proportion of a teaspoonful of brandy in a tablespoonful of water, or a little aromatic spirits of ammonia, ten drops every few minutes in a tablespoonful of water.

